# Gut microbiota profile and atopic dermatitis in the first year of life

**DOI:** 10.25122/jml-2024-0287

**Published:** 2024-10

**Authors:** Alexandru Cosmin Pantazi, Wassan Nori, Mustafa Ali Kassim Kassim, Adriana Luminita Balasa, Cristina Maria Mihai, Tatiana Chisnoiu, Larisia Mihai, Adina Ungureanu, Corina Elena Frecus, Sergiu Ioachim Chirila, Simona Claudia Cambrea

**Affiliations:** 1 Faculty of Medicine, Ovidius University, Constanta, Romania; 2 Department of Pediatrics, Clinical Emergency Hospital of Constanta, Constanta, Romania; 3 College of Medicine, Mustansiriyah University, Baghdad, Iraq

**Keywords:** infants, atopic dermatitis, gut microbiota, dysbiosis, proteolytic bacteria, acidifying bacteria

## Abstract

The connection between the immune response and the composition of gut microbiota has been associated with an increased prevalence of atopic dermatitis in the first year of life. The study aimed to investigate gut microbiota characteristics in infants with atopic dermatitis compared to healthy infants to better understand the link between early-life microbiota composition and the development of atopic dermatitis. The study analyzed the intestinal microbiota of 121 infants with clinical signs of atopic dermatitis, divided into Group I (infants with atopic dermatitis) and Group II (healthy controls). The study showed that infants with atopic dermatitis presented increased values of proteolytic bacteria mainly represented by *Enterobacter* species (*P* = 0.041), *Klebsiella* species (*P* = 0.038), and *Escherichia coli* (*P* = 0.013), with significantly decreased levels of acidifying bacteria represented by *Enterococcus* species, *Lactobacillus* and *Bifidobacterium* (*P* < 0.05) and normal levels of *Clostridium* species, *Candida albicans, Mould fungi* and *Geotrichum* species. This study highlights distinct differences in the gut microbiota of infants with atopic dermatitis, providing insights into the dynamic intestinal ecosystem during early life for future personalized therapeutic strategies.

## INTRODUCTION

Atopic dermatitis is a common skin condition frequently observed in children, caused by a combination of hereditary and environmental factors [1], substantially affecting the quality of life [2]. The incidence of atopic dermatitis in children is increasing worldwide [3]. Implications of gut microbiota in allergies and atopic dermatitis were reported by previous studies [4], emphasizing the complex relationship between gut microbiota composition and human health, which justifies the need for additional research in the field of personalized medicine [5].

Infants are highly prone to developing atopic dermatitis, often experiencing more frequent and severe flare-ups that tend to last longer [6]. They are also more likely to develop the condition at an earlier age [6]. Exposure to a food antigen can trigger a hypersensitive skin reaction, which may lead to a food allergy [7]. This highlights the significant connection between the skin and the immune response in the gut [7]. The gut microbiota plays a crucial role in autoimmune disorders [8,9] and infectious diseases [10], with long-lasting effects. Novel therapeutics targeting the gut microbiota have shown promising results [8,11].

Previous studies have demonstrated that modifying the gut microbiota can potentially impact the immune response [12,13] and have also indicated a connection between changes in gut microbiota and the development of allergy disorders [14]. Melli *et al*. [15] found that the gut microbiota composition differs between allergic and non-allergic children, suggesting that alterations in intestinal microbiota may be associated with the onset of atopic dermatitis symptoms. Kong *et al*. [16] also reported changes in microbiota composition throughout the progression of atopic dermatitis.

Gut dysbiosis in infancy has been linked to immune system development and often precedes the onset of atopic diseases, with atopic dermatitis being the initial phase of the atopic march [17]. Furthermore, microbiota patterns in the skin and gut can predict susceptibility to dietary and exterior allergens or trigger allergic reactions in the host [18].

Certain microorganisms are associated with infectious processes [19,20] due to the influence of environmental variables [21], while changes in the gut microbiota composition may contribute to the development of atopic dermatitis [22]. The existing therapeutic strategies for atopic dermatitis are limited by a lack of effective options and the heterogeneous nature of the illness [23].

Recent studies have emphasized the importance of modulating gut microbiota and its implications for allergies [14], respiratory diseases [24], and renal conditions [25], highlighting the long-term impact on overall health [26]. Modulating gut microbiota has emerged as a promising treatment strategy for infants with atopic dermatitis, demonstrating encouraging results [27]. Identifying the composition and characteristics of intestinal microbiota in infants with atopic dermatitis represents a key factor for future personalized therapy.

This study aimed to investigate gut microbiota variations in infants with atopic dermatitis compared to healthy infants. These findings reveal insights into the connection between the composition of the dynamic intestinal ecosystem and atopic dermatitis for future personalized therapeutic strategies.

## MATERIAL AND METHODS

The study was conducted from April 2023 to May 2024 and included 121 infants diagnosed with atopic dermatitis. The infants were divided into two groups: Group I consisted of 91 infants with atopic dermatitis (AD), and Group II (the control group) included 30 infants without atopic dermatitis (non-AD). Demographic data were collected, and informed consent was obtained from the legal representatives of all participants for both participation and the processing of personal data. The study adhered to the ethical principles outlined in the Declaration of Helsinki.

Participants were selected based on the following criteria: age between 1 month and 1 year, a confirmed diagnosis of atopic dermatitis, and the absence of acute infectious diseases, gastrointestinal disorders, cardiovascular diseases, renal diseases, endocrine disorders, oncological conditions, autoimmune diseases, and the use of probiotics or antibiotics within four weeks before the study.

The diagnosis of AD was assessed by the dermatologist based on the presence of distinct physical characteristics and patterns of skin lesions, a persistent or recurring course, itching, and a personal or family history of atopic conditions, as outlined in the Williams criteria [28] and the Hanifin and Rajka criteria [28, 29].

Exclusion criteria included infants older than 1 year, those with gastrointestinal or genetic disorders, endocrine or metabolic diseases, blood disorders, heart, liver, or kidney conditions, recent use of antibiotics (within one month), or probiotics (within 4 weeks).

Stool samples were collected to determine the composition of gut microbiota. Fecal samples were collected during clinic visits, stored at -2°C, and promptly transported to the laboratory in sterile containers. A total of 1 g of each sample was used for bacteriological and fungal analysis. Bacteriological examination of stool samples was performed for proteolytic bacteria (*Escherichia coli, Proteus* species, *Klebsiella* species, *Enterobacter* species, *Hafnia alvei, Serratia* species, *Providencia* species, *Morganella morganii, Kluyvera* species, *Citrobacter* species, *Pseudomonas* species, *Clostridium* species) and acidifying bacteria (*Bacteroides* species, *Bifidobacterium* species, *Lactobacillus* species, *Enterococcus* species) and for fungal species (*Candida albicans, Candida* species, *Geotrichum* species and *Mould fungi*). The plates were incubated in optimal growth conditions of the target species. To facilitate understanding, we categorized the identified genera based on their abundance (high or low) in infants with atopic dermatitis.

Results were analyzed using descriptive statistics. Standard deviation (SD) was employed to measure the variability of continuous data, and a *P*-value of 0.05 was considered statistically significant. The reliability of differences between comparison groups was assessed using the *t*-test.

## RESULTS

The breastfeeding rate in the atopic dermatitis (AD) group was lower compared to the control group. The analysis of the type of delivery revealed that cesarean section had a higher occurrence in the AD group. The infants were born at full-term with an average weight of 2.98 kilograms (SD = 0.24) and an average height of 50.21 centimeters (SD = 1.43). The general characteristics of the two groups are presented in Table 1.

**Table 1:** Characteristics of participants

	AD group	Non-AD group
Male, *n* (%)	43 (47.3)	14 (46.7)
Female, *n* (%)	48 (52.7)	16 (53.3)
Age (months) mean (SD)	7.62 (1.7)	6.84 (1.8)
Vaginal delivery, *n* (%)	40 (43.9)	17 (56.6)
Cesarean section, *n* (%)	51 (56.1)	13 (43.4)
Breastfeeding, *n* (%)	40 (43.9)	19 (63.4)
Formula feeding, *n* (%)	35 (38.5)	7 (23.3)
Mixed feeding (infant formula and breastfeeding), *n* (%)	16 (17.6)	4 (13.3)
Age at onset of AD, months (mean)	5.12	-
Relapse episodes of AD (mean)	2.08	-
Family history of atopy		
One parent, *n* (%)	68 (74.7)	11 (36.6)
Both parents, *n* (%)	23 (25.3)	4 (13.3)

The fecal microbial analysis identified significant alterations in the intestinal microbiota of infants with atopic dermatitis (Table 2). The atopic dermatitis group had a higher abundance of *Proteobacteria phylum*, mainly represented by gram-negative, facultatively anaerobic *Enterobacteriaceae* family. There were also low concentrations of *Firmicutes phylum* and *Actinobacteria phylum*. The presence of *Bacteroidetes phylum* was significantly higher in the AD group (*P* = 0.036), with concentrations of 2.31 ± 0.08 x 10^9^ CFU/g.

**Table 2:** Composition of intestinal microbiota in AD group and non-AD group

Microorganisms (CFU/g)	AD group (*n* = 91)	Non-AD group (*n* = 30)
*Escherichia coli* (x10^8^)	1.8 ± 0.4 (*P* = 0.013)	0.8 ± 0.32
*Proteus* spp. (x10^4^)	0.83 ± 0.12 (*P* = 0.611)	0.89 ± 0.04
*Klebsiella* spp. (x10^4^)	1.32 ± 0.3 (*P* = 0.038)	0.85 ± 0.06
*Enterobacter* spp. (x10^4^)	1.12 ± 0.2 (*P* = 0.041)	0.83 ± 0.12
*Hafnia alveii* (x10^4^)	0.91 ± 0.02 (*P* = 0.311)	0.95 ± 0.03
*Serratia* species (x10^4^)	0.90 ± 0.02 (*P* = 0.218)	0.92 ± 0.06
*Providencia* species (x10^4^)	0.83 ± 0.03 (*P* = 0.183)	0.89 ± 0.05
*Morganella morganii* (x10^4^)	0.84 ± 0.02 (*P* = 0.164)	0.88 ± 0.04
*Kluyvera* species (x10^4^)	0.90 ± 0.02 (*P* = 0.913)	0.91 ± 0.07
*Citrobacter* species (x10^4^)	0.78 ± 0.04 (*P* = 0.875)	0.95 ± 0.13
*Pseudomonas species* (x10^4^)	0.92 ± 0.02 (*P* = 0.213)	0.96 ± 0.02
*Clostridium* species (x10^5^)	0.85 ± 0.05 (*P* = 0.098)	0.94 ± 0.03
*Bacteroides* species (x10^9^)	2.31 ± 0.08 (*P* = 0.036)	0.94 ± 0.04
*Bifidobacterium* species (x10^8^)	0.75 ± 0.08 (*P* = 0.038)	0.93 ± 0.06
*Lactobacillus* species (x10^5^)	0.82 ± 0.05 (*P* = 0.021)	0.90 ± 0.07
*Enterococcus* species (x10^5^)	0.71 ± 0.02 (*P* = 0.042)	0.92 ± 0.03
*Candida albicans* (x10^3^)	0.80 ± 0.08 (*P* = 0.930)	0.96 ± 0.02
*Candida* species (x10^3^)	0.91 ± 0.04 (*P* = 0.221)	0.88 ± 0.08
*Geotrichum* species (x10^3^)	0.89 ± 0.03 (*P* = 0.135)	0.90 ± 0.07

**Figure 1:**
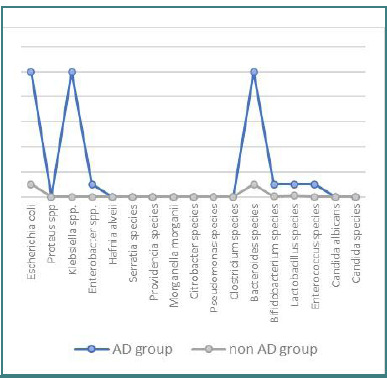
Composition of intestinal microbiota in AD and non-AD groups

The findings revealed a higher prevalence of intestinal dysbiosis in the AD group, with increased concentrations of proteolytic bacteria, including *Escherichia coli* (1.8. ± 0.4 x 10^8^ CFU/g, *P* = 0.013), *Enterobacter* species (1.12 ± 0.2 x 10^4^ CFU/g, *P* = 0.041), and *Klebsiella* species (1.32 ± 0.3 x 10^4^ CFU/g, *P* = 0.038). There was also a significant increase in *Bacteroides* species among acidifying bacteria (2.31 ± 0.08 x 10^9^ CFU/g, *P* = 0.036) in the AD group compared to the control group (Figure 1).

Infants with atopic dermatitis had significantly lower levels of acidifying germs represented by *Bifidobacterium, Lactobacillus*, and *Enterococcus*, compared to the control group (*P* < 0.05). *Candida albicans* and *Geotrichum* species had normal concentrations in both groups, and no mold fungi were detected.

The quantitative analysis showed lower *Bifidobacterium* spp. levels in the AD group (0.75 ± 0.08 x 10^8^ CFU/g, *P* = 0.038) compared to the non-AD group (0.93 ± 0.06 x 10^8^ CFU/g). *Lactobacillus* spp. concentrations were also reduced in the AD group (0.82 ± 0.05 x 10^5^ CFU/g, *P* = 0.021) compared to the non-AD group (0.90 ± 0.07 x 10^5^ CFU/g). *Enterococcus* spp. levels were significantly lower in the AD group (0.71 ± 0.02 x 10^5^ CFU/g, *P* = 0.042) compared to the non-AD group (0.92 ± 0.03 x 10^5^ CFU/g).

**Figure 2:**
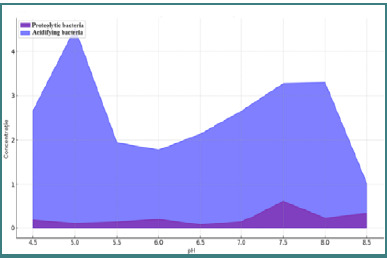
Concentration of acidifying and proteolytic bacteria according to fecal pH values

Normal values were observed for *Hafnia alvei, Citrobacter* species, *Serratia* species, *Providencia* species, *Pseudomonas* species, *Clostridium* species, and *Morganella morganii* in both groups. An increase in proteolytic bacteria concentration was associated with a more alkaline fecal pH (mean value 6.5), indicating a preference for these bacteria in higher pH environments. Conversely, acidifying bacteria thrived at lower pH values. Figure 2 shows the acidifying and proteolytic bacteria concentration according to fecal pH values.

## DISCUSSION

The study found significantly decreased levels of acidifying germs, such as *Bifidobacterium* spp., in infants with AD compared to healthy infants at the phylum level, with a high abundance of *Bacteroides* species. Abrahamsson *et al*. [30] reported reduced levels of *Bacteroidetes* associated with atopic dermatitis and decreased levels of *Proteobacteria* at one year. Additional research demonstrated a positive correlation between the incidence of AD and increased abundance of *Bacteroidaceae* and *Bacteroides* [31].

An increased abundance of *Bacteroides* has been linked to atopic manifestations, potentially resulting in continuous lipopolysaccharide synthesis and triggering inflammatory responses [32,33]. *Bacteroides* species have also been found to alter gut permeability, a characteristic observed in individuals with atopic dermatitis [32]. Additional studies showed that a high abundance of *Bacteroides* species and proteolytic bacteria, such as *Escherichia coli* and *Enterobacter* species, contribute to the onset of eczema during the first year of life [33,34].

Infants with atopic dermatitis presented significantly decreased levels of acidifying germs represented by *Bifidobacterium, Lactobacillus*, and *Enterococcus*, compared to the control group. Additional studies highlighted the association between the gut microbiota composition of healthy infants and AD, with a decrease in the presence of *Bifidobacterium* and *Lactobacillus*, particularly in infants with atopic dermatitis [35]. Chen *et al*. [36] reported an increased level of *Firmicutes* with a lower concentration of *Bacteroidetes* and increased values of *Bifidobacterium, Enterococcus, Lactobacillus, Roseburia, Faecalibacterium, Ruminococcus*, and *Akkermansia* in children with allergies.

Hong *et al*. [37] showed that infants with atopic dermatitis presented elevated concentrations of *Klebsiella* spp., the main phyla represented by *Firmicutes, Proteobacteria, Actinobacteria*, and *Bacteroidetes*. The abundance of *Bifidobacterium* and *Enterococcus* species was also linked to the occurrence of AD in infants delivered via cesarean section [37,38]. Our study findings revealed increased values of proteolytic germs represented by *Escherichia coli* (*P* = 0.013), *Enterobacter* species (*P* = 0.041), and *Klebsiella* species (*P* = 0.038).

A significant correlation between higher levels of *Clostridiaceae* and an increased risk of AD has been reported [34]. This association may be attributed to the release of toxins that hinder the movement of neutrophils and deactivate eosinophils, thereby exacerbating inflammation [34]. Atopic infants were shown to have elevated levels of *Clostridium* and reduced levels of *Bifidobacterium* in their stools [39]. Our study, however, showed normal concentrations of *Clostridium* species in infants with AD.

*Parabacteroides* and *Klebsiella* were more prevalent in infants with atopic dermatitis [40]. Our study provides additional evidence for this observation by noting a higher concentration of *Klebsiella, Escherichia coli*, and *Enterobacter* in the AD group.

Regarding fungal species, *Candida albicans* and *Geotrichum* species were found at normal concentrations in both the AD and non-AD groups. However, other studies have reported a link between high levels of *Candida albicans* and an increased risk of AD in the first year of life [41,42].

Enhanced therapeutic outcomes can be achieved by integrating microbiota-based therapies with conventional treatments. Recent studies focus on treatments such as multi-strain probiotics, prebiotics, and fecal microbiota transplantation [43].

Long-term intake of probiotics can modify the gut microbial habitat and maintain a healthy balance of gut microbiota and systemic immune responses [44]. Probiotics enhance the level of short-chain fatty acids (SCFA) in the intestinal lumen [44]. Specifically, short-chain fatty acids (SCFA) such as acetate, propionate, and butyrate create an intestinal environment with a low pH and compete with pathogens, suppressing excessive growth [44]. Hence, probiotics can potentially mitigate the clinical symptoms of AD by influencing the makeup of gut microbiota, metabolic processes, and immunological reactions [44]. The preventive impact of *Lactobacillus rhamnosus* and mixed probiotics is beneficial due to the decreased abundance of *Bifidobacterium* and *Lactobacillus* species in infants with atopic dermatitis [45]. Some studies have suggested administering probiotics, specifically *Lactobacillus* or *Bifidobacterium* strains, to pregnant women in the last trimester to prevent AD in infants [46]. Additionally, administering probiotics and vitamin D supplements during pregnancy and infancy while minimizing needless antibiotic use may decrease the risk of atopic dermatitis [46].

Novel technologies such as CRISPR-based genome editing and high-throughput screening could improve therapeutic capabilities on probiotic strains [47]. Microencapsulation techniques improve the stability, viability, and targeted distribution of probiotics, enhancing their effectiveness [47].

Fecal microbiota transplantation (FMT) is a new therapeutic approach used to restore the gut microbiota. The therapeutic potential of FMT was examined through the alteration of gut microbiota, modulation of the immune system, and measurement of fecal metabolites in an AD mice model [48], emphasizing the necessity for additional research in this area.

The current study is subject to significant limitations. Firstly, it did not examine stool samples before the onset of AD, hence impeding the establishment of a temporal relationship. It is important to note that intestinal microbiota composition might differ significantly across individuals. In this particular age group, the intestinal flora is still undergoing active and ongoing development. Furthermore, the description of potential confounding factors was inadequate due to the omission of some influential factors that impact the gut microbiota, such as maternal antibiotic exposure.

## CONCLUSION

The study reveals characteristics in the composition of gut microbiota in infants with atopic dermatitis, with specific variation in acidifying and proteolytic germs, with higher diversity of proteolytic bacteria represented by *Escherichia coli*, and increased colonization of *Enterobacter* spp. and *Klebsiella* spp., with a lower diversity of acidifying germs represented by *Lactobacillus, Enterococcus* and significantly decreased values of *Bifidobacterium* spp. The study shows distinct variations of gut microbiota in the genus characteristics in infants with atopic dermatitis compared to healthy infants. This research provides valuable insights into the relationship between intestinal bacterial composition and atopic dermatitis, paving the way for future therapeutic strategies in personalized medicine.
